# Leptospirosis in Coastal South India: A Facility Based Study

**DOI:** 10.1155/2018/1759125

**Published:** 2018-05-15

**Authors:** Ramesh Holla, Bhagwan Darshan, Latika Pandey, Bhaskaran Unnikrishnan, Nithin Kumar, Rekha Thapar, Prasanna Mithra, Vaman Kulkarni

**Affiliations:** ^1^Department of Community Medicine, Kasturba Medical College, Manipal Academy of Higher Education, Mangalore 575001, India; ^2^Kasturba Medical College, Manipal Academy of Higher Education, Mangalore 575001, India

## Abstract

**Background:**

Leptospirosis is a major neglected public health problem and is highly underreported in India. The spectrum of clinical features ranges from subclinical infection to multiorgan failure. The burden of leptospirosis is more in developing countries.

**Objective:**

The present study was designed to assess the sociodemographic characteristics, clinical feature, and outcome of leptospirosis patients.

**Methods:**

This record based retrospective study was conducted in hospitals affiliated to Kasturba Medical College, Mangalore. The registries of the leptospirosis patients during the period of four years between January 2011 and December 2014 were reviewed and the information on demographic and clinical profile of the leptospirosis patients was recorded in the data capture sheet. The information obtained was analyzed using SPSS version 11.5.

**Results:**

The study included 202 leptospirosis victims. The mean age of the study population was 40.48 (±14.8) years. Majority of the patients presented with fever (92.1%) at the time of admission. Acute renal failure was found to be the most common complication (79.2%). Case fatality rate was found to be 3.5%.

**Conclusion:**

High proportion of cases indirectly reflects the endemic nature of the disease in the study setting. The clinical presentation of the leptospirosis is highly protean and may vary from a mild illness to life-threatening complications as evident from the current study.

## 1. Introduction

Leptospirosis is essentially a zoonotic disease that is caused by spirochetes of the genus* Leptospira* and is prevalent worldwide [1]. It is an infectious disease caused by pathogenic strains of* Leptospira* species, of which almost 20 serogroups and 200 serovars are currently known. The problem of the disease has not been completely addressed even though it has been recognized for decades, the primary reason being the presence of the major burden of the disease in poor, developing countries [2]. Leptospirosis has been recognized as major public health problem and multiple epidemics have been reported, owing to the occurrence of natural disasters and the prevalence of poor sanitary conditions [3, 4]. Very little is currently known regarding the true incidence of leptospirosis. However, it is estimated that 10 or more per 100,000 people are affected with this disease each year in tropical climates. If there is an epidemic, the incidence can soar to 100 or more per 100,000 people [3]. While humans are accidental hosts, the primary reservoir hosts include infected animals such as rodents, dogs, cattle and pigs, and infection is acquired by humans through direct or indirect contact with water or soil contaminated by the urine of infected animals [5].

Important epidemiological risk factors in the occurrence of the disease include contaminated environment and rainfall [6]. Children acquire the infection from dogs more commonly than do adults. Occupational exposure is a major cause of infection and the risk groups include agricultural and livestock farmers, workers in underground sewers, meat and animal handlers, and veterinarians [4].

The spectrum of the disease ranges from subclinical infection to a severe syndrome characterized by multiorgan dysfunction. Clinical features include headache, fever, myalgia, jaundice, conjunctival suffusion, bleeding tendencies, oliguria, and pulmonary manifestations like cough, breathlessness, and hemoptysis [7]. The mild, anicteric form of the disease is more common and presents with nonspecific symptoms while the icteric form of the disease is potentially fatal and presents with jaundice, renal dysfunction, and bleeding diathesis [8]. In many cases, leptospirosis can present without any classical features [5]. Laboratory investigations are essential for the confirmation of disease as vague clinical symptoms make the diagnosis difficult [7]. Common complications of the disease include renal failure, respiratory failure, neuroleptospirosis, and Disseminated Intravascular Coagulation [8]. Leptospirosis is highly underreported in India, most likely due to lack of diagnostic modalities and lack of awareness among clinicians [9]. It is important to diagnose the disease timely as early initiation of antibiotic therapy is highly beneficial in interrupting the course of the disease [10].

The burden of leptospirosis is more in developing countries and there is paucity of literature available on the burden and varied clinical manifestations of this disease in India. The present study is being undertaken in one of the coastal districts of Southern India where agriculture-related activities form a major occupation of the population and are at risk of acquiring leptospirosis. The present study was designed to assess the sociodemographic characteristics, clinical feature, and outcome of leptospirosis patients.

## 2. Materials and Methods

The present registry-based retrospective study was carried out among all patients admitted with a diagnosis of leptospirosis at the hospitals affiliated to Kasturba Medical College, Mangalore. It acts as a referral center for coastal part of Karnataka and northern parts of Kerala. Informed consent was not obtained as it was a retrospective case record study; however, approval was taken from the Institutional Ethics Committee (IEC) of Kasturba Medical College, Mangalore (Manipal Academy of Higher Education), before commencement of the study. Permission was then obtained from the department of General Medicine and Medical Superintendent of the hospital to access the registries of leptospirosis patients from the medical records department. The registries of the leptospirosis patients during the period of four years between January 2011 and December 2014 were reviewed and the information on demographic and clinical profile of the leptospirosis patients seeking healthcare (only serologically confirmed cases through Lepto IgM ELISA) was recorded in the data capture sheet. The information obtained was analyzed using SPSS (Statistical Package for Social Sciences) version 11.5 for descriptive statistics, and the results were expressed in proportions, mean, and standard deviation.

## 3. Results

The baseline characteristics of leptospirosis patients are displayed in Table 1. The mean age of the study population is 40.48 (±14.8) years and the age ranged from 11 to 90 years. Majority of the leptospirosis victims were in the age group of 20–40 years (*n* = 80, 39.6%) and 41–60 years (*n* = 82 40.6%), thus affecting the working population. It was observed that majority of the victims were males (*n* = 142, 70.3%). When the duration of the hospital stay was analyzed, it was seen that most of the victims were discharged from the hospital within 5 days (*n* = 94, 46.5%). However the median duration of stay in the hospital was 6.0 days [IQR: 4–9 days], ranging from 1 to 23 days.

Table 2 shows the distribution of leptospirosis patients according to clinical presentation at the time of admission. Majority of the patients presented with fever (*n* = 186, 92.1%) at the time of admission followed by myalgia and generalized weakness (*n* = 73, 36.1%) and vomiting (*n* = 66, 32.7%).

The laboratory profile of most of the patients was consistent with elevated serum bilirubin levels (*n* = 118, 76.6%) followed by elevated AST levels (*n* = 116, 73.4%) and thrombocytopenia (*n* = 113, 71.5%). The mean Hb value was 11.6 (±2.5) gm% and ranged from 4.7 to 18.5 gm%. The median total leukocyte count was 11,300 [IQR: 7350–17825]. The total leukocyte count ranged from 800 to 77000. Among the leptospirosis victims who were investigated for renal parameters, it was seen that more than half of them had elevated urea (63.9%) and creatinine levels (53%) as depicted in Table 3.

When the clinical outcome of the leptospirosis patients was analyzed, it was observed that most of the patients recovered without any complications (*n* = 151, 74.7%) and nearly one-fifth of them recovered with complications (*n* = 44, 21.8%). When the type of complications was further analyzed it was noted that acute renal failure was the most commonly seen (*n* = 35, 79.2%). The case fatality was found to be 3.5% as observed in Table 4.

## 4. Discussion

Leptospirosis is prevalent worldwide but is most commonly seen in tropical and subtropical regions due to excessive rain and flooding. It is often transmitted through water and food contaminated by urine of infected rats whereas human to human transmission is rare [2]. A hospital based case record study was carried out in leptospirosis patients to determine the sociodemographic profile, symptomatology, and the outcome of the disease.

Mean age of the leptospirosis patients was found to be 40.4 years in the present study, while in a study done in Maharashtra it was found to be 42 years [10]. This is in congruence with the findings recorded in studies conducted at different parts of Northern India [7–9, 11]. A study done at southern part of India to analyze the changing pattern of leptospirosis patients revealed that though the people of all age group were affected, maximum number was observed in adulthood because of their work pattern [6]. In line with our study findings, people of productive age group were affected in other studies conducted in different parts of South India [12–14] and central India [15], thus imparting economic misery to the affected families.

Predomination of males over females were observed among leptospirosis cases in studies conducted at Ludhiana [7], Thirupathi [12], Mangaluru [13], and Chennai [14] which is similar to the present study wherein three-fourths of the victims were males. In contrast to a study conducted at Maharashtra where the mean duration of hospital stay was sixteen days, the duration in the present study was observed to be eleven days [10].

Leptospirosis is febrile systematic disease and symptoms manifested depend on the organs involved. It mainly involves central nervous system, reproductive system, liver, lung, eyes, kidney, and reproductive system [16]. Fever was the most common clinical presentation in the present study which was seen among more than four-fifths of the leptospirosis patients. This is consistent with the findings observed in the studies conducted at different parts of northern [8, 10] and Southern India [12–14] and sub-Himalayan region [11]. A study conducted at western Maharashtra has revealed jaundice as the most common symptom which was noted among three-fourths of the leptospirosis patients [10], whereas one-fifth of the patients were presented with jaundice in the current study. Myalgia was seen in almost 40% of the patients at the time of admission which was similar to the observation made in a study conducted in Chandigarh [8]. Other common symptoms at the time of admission include headache, vomiting, and oliguria. This is similar to results of various studies which are carried out in North India, Kolkata, and Punjab [7, 9, 17]. Respiratory symptoms were seen in 10% of the patients in the current study which is comparable to that reported by a study done at North India [8].

Laboratory findings are essential to confirm a case of leptospirosis as clinical manifestation of leptospirosis is nonspecific [16]. The case definitions of leptospirosis, that is, suspect, probable, and confirmed, have been followed for managing the case in our hospitals. Serologically the cases were confirmed by doing Lepto IgM ELISA. On recording the laboratory parameters, it was observed that more than half of the patients had total leukocyte count of more than 11000. This is similar to the results observed in a study conducted in Chandigarh [8]. Hemoglobin values of less than 11 gm% were recorded in one-third of the patients whereas in a study conducted in Ludhiana only one-tenth of the patients had anemia [7]. Thrombocytopenia was seen in almost three-fourths of the study population. This is in concordance with a study which was carried out in Kolkata [17]. Most of the patients were found to have elevated liver enzymes and increased bilirubin levels which is consistent with various other studies conducted on leptospirosis patients [7, 9, 10]. Serum creatinine values of more than 1.4 mg/dl were found in more than half of the patients in the present study while a study done at North India observed increased creatinine levels in less than 10% of the patients [7].

Complications of leptospirosis include pulmonary hemorrhage, renal failure, icterus, myocarditis, and uveitis [16]. It was evident from our study that acute renal failure was the most common complication seen in leptospirosis patients. Studies which were conducted in western Maharashtra [5], Northern India [10], and southern part of India [12, 14] have also stated the same. The rarer complications encountered in our study included ARDS (Acute Respiratory Distress Syndrome), dilated cardiomyopathy, sepsis, and multiorgan dysfunction syndrome. In a study done at Chandigarh, hemorrhagic pneumonia, neuroleptospirosis, and Disseminated Intravascular Coagulation were seen as rarer complications [8]. In the same context, a study done at western Maharashtra showed meningoencephalitis, Deep Vein Thrombosis, and hepatic encephalopathy as few of the rare complications seen in leptospirosis patients [10].

Case fatality rate for leptospirosis can be as high as 30% [18]. Present study showed that most of the patients recovered without any complications while the case fatality rate was only 3.5% which is comparable to the study done at Punjab (5.9%) [9]. However higher case fatality rate was observed in a study conducted at coastal part of South India [13]. The lesser case fatality rate in the present study can be attributed to dialysis for renal failure patients and good nursing care at the study setting. On the other hand, study done at sub-Himalayan region of North India did not encounter any deaths in their study population [11].

## 5. Conclusion

Leptospirosis remains a significant public health problem mainly affecting the population of productive age group. High proportion of cases indirectly reflects the endemic nature of the disease in the study setting. The clinical presentation of the leptospirosis is highly protean and may vary from a mild illness to life-threatening complications as evident from the current study. As the disease is endemic and can have a fatal outcome, it should raise a high index of suspicion among the clinical fraternity when they come across a patient with fever and jaundice. A well planned multicentric study done at different geographical locations could bring out better insight to the epidemiology of leptospirosis.

## Figures and Tables

**Table 1:** Baseline characteristics of leptospirosis patients (*N* = 202).

Baseline characteristics	Number	Percentage
*Age group (years)*		
<20	022	10.9
20–40	080	39.6
41–60	082	40.6
>60	018	08.9
*Sex*		
Male	142	70.3
Female	060	29.7
*Religion*		
Hindu	191	94.5
Muslim	007	03.5
Christian	004	02.0
*Hospital*		
Government	121	59.9
Private	081	40.1
*Duration of stay (days)*		
<5	094	46.5
6–10	073	36.1
>10	035	17.4

**Table 2:** Distribution pattern of the chief presenting complaints (*N* = 202).

Clinical presentation	Number^*∗*^	Percentage
Fever	186	92.1
Myalgia and generalized weakness	073	36.1
Vomiting	066	32.7
Fever with chills and rigors	054	26.7
Oliguria	052	25.7
Jaundice	045	22.3
Abdominal pain	038	18.8
Headache	037	18.3
Cough	021	10.4
Conjunctival suffusion	009	04.5

^**∗**^Multiple responses.

**Table 3:** Laboratory profile of leptospirosis patients.

Lab parameter	*N*	*n* (%)^**∗**^
Anemia (Hb < 11 gm%)	159	059 (37.1)
Leukocytosis (TC > 11000)	164	083 (50.6)
Neutrophilia (*N* > 70%)	143	095 (66.4)
Lymphopenia (*L* < 20%)	140	096 (68.6)
Thrombocytopenia (<1.5 lakhs)	158	113 (71.5)
Serum hyperbilirubinemia (>1.2 mg/dl)	162	109 (67.7)
Serum direct bilirubin > 0.2 mg/dl	154	118 (76.6)
Serum urea > 45 gm/dl	156	099 (63.9)
Serum creatinine > 1.4 mg/dl	168	088 (53.0)
Serum hypoproteinemia (TP < 6.0 gm/dl)	139	074 (53.2)
Serum hypoalbuminemia (Alb < 3.2 gm/dl)	143	087 (60.8)
*Elevated liver enzymes*		
AST (>40 units)	158	116 (73.4)
ALT (>40 units)	160	098 (61.2)
ALP (>129 units)	147	074 (50.7)

^**∗**^Multiple responses.

**Table 4:** Clinical outcome of leptospirosis patients (*N* = 202).

Outcome	Number	Percentage
Recovered without complications	151	74.7
Recovered with complications	044	21.8
Death	007	03.5

## Data Availability

The data used to support the findings of the present study is available and will be made available by the corresponding author upon request.

## References

[1] Longo D., Fauci A. S., Kasper D. L., Hauser S., Jameson J., Loscalzo J. (2012). Harrisons principles of internal medicine.

[2] Victoriano A. F. B., Smythe L. D., Gloriani-Barzaga N. (2009). Leptospirosis in the Asia Pacific region.

[3] World Health Organisation The Global Burden of Leptospirosis. http://www.who.int/zoonoses/diseases/lerg/en/index2.html.

[4] Park K. (2011).

[5] Karande S., Bhatt M., Kelkar A., Kulkarni M., De A., Varaiya A. (2003). An observational study to detect leptospirosis in Mumbai, India, 2000.

[6] Shivakumar S. (2006). Leptospirosis in chennai - Changing clinical profile [3].

[7] Deodhar D., John M., Leptospirosis. (2011). Experience at a tertiary care hospital in northern India.

[8] Sethi S., Sood A., Pooja Sharma S., Sengupta C., Sharma M. (2003). Leptospirosis in northern india: a clinical and serological study.

[9] Sethi S., Sharma N., Kakkar N. (2010). Increasing trends of leptospirosis in Northern India: a clinico-epidemiological study.

[10] Patil H., Agrawal V., Patil V. (2012). Clinical profile and outcome of leptospirosis at tertiary care centre in western Maharashtra.

[11] Chauhan V., Mahesh D. M., Panda P., Mokta J., Thakur S. (2010). Profile of patients of leptospirosis in sub-Himalayan region of north India.

[12] Thalva C., Desamani K. K. (2017). Socio-demographic, clinical, epidemiological and laboratory profile of cases of leptospirosis at tertiary care hospital: a two year study.

[13] Mansoor S. M., Hemant K., Jayram S., Poojari. (2005). A clinic epidemiological profile of cases of Leptospirosis in a tertiary care hospital.

[14] Arumugam M., Ganesan R., Sameem Khan I., Kalaiselvan V. (2016). A study on clinical profile and complications of leptospirosis at kilpauk medical college, chennai.

[15] Mavatkar M. V., Sujatha P., Singh V., Gokhe S B. (2017). A study of five years trend of Case Fatality Rate of Leptospirosis in a tertiary care hospital in Mumbai.

[16] National Institute of Communicable Diseases (2005).

[17] Datta S., Sarkar R. N., Biswas A., Mitra S. (2011). Leptospirosis, an institutional experience.

[18] World Health Organization-International Leptospirosis Society (2003). http://www.who.int/csr/don/en/WHO_CDS_CSR_EPH_2002.23.pdf.

